# Can one puff really make an adolescent addicted to nicotine? A critical review of the literature

**DOI:** 10.1186/1477-7517-7-28

**Published:** 2010-11-10

**Authors:** Reuven Dar, Hanan Frenk

**Affiliations:** 1 Tel Aviv University, P.O. Box 39040, Tel Aviv 69978, Israel; 2 The Academic College of Tel Aviv-Yafo, P.O. Box 16131, Tel Aviv, Israel

## Abstract

**Rationale:**

In the past decade, there have been various attempts to understand the initiation and progression of tobacco smoking among adolescents. One line of research on these issues has made strong claims regarding the speed in which adolescents can become physically and mentally addicted to smoking. According to these claims, and in contrast to other models of smoking progression, adolescents can lose autonomy over their smoking behavior after having smoked one puff in their lifetime and never having smoked again, and can become mentally and physically "hooked on nicotine" even if they have never smoked a puff.

**Objectives:**

To critically examine the conceptual and empirical basis for the claims made by the "hooked on nicotine" thesis.

**Method:**

We reviewed the major studies on which the claims of the "hooked on nicotine" research program are based.

**Results:**

The studies we reviewed contained substantive conceptual and methodological flaws. These include an untenable and idiosyncratic definition of addiction, use of single items or of very lenient criteria for diagnosing nicotine dependence, reliance on responders' causal attributions in determining physical and mental addiction to nicotine and biased coding and interpretation of the data.

**Discussion:**

The conceptual and methodological problems detailed in this review invalidate many of the claims made by the "hooked on nicotine" research program and undermine its contribution to the understanding of the nature and development of tobacco smoking in adolescents.

## Review

Anthony et al. [1] observed that most teenagers (75.6%) experiment with tobacco but less than one third of those (31.9%) develops tobacco dependence. This finding raises two important questions, which have received considerable attention in smoking research over the past decades. First, what drives adolescents to experiment with smoking? Second, why do a sizeable proportion of these youngsters become habitual and heavy smokers in spite of the widely publicized health hazards associated with smoking?

Most researchers believe that the answers to these questions are complex and partially overlapping. Both the latency to the first puff and subsequent progression to daily smoking are correlated with a variety of parameters, including gender [2], sociostructural [3] and socioeconomic [2,4-6] variables, early dating [7], personality variables [8], parental [9,10] and peer smoking [2,11], disorderly conduct [4-6,10], academic achievement [11], ethnicity [2], self-efficacy [2], mental health [4-6,12], religiosity [13], restaurant smoking restrictions [14], and use of other drugs [4,5,15]. In addition, several studies have postulated that progression to regular smoking is associated with a positive experience with the first cigarette. Evidence for this hypothesis comes from studies of smokers' and nonsmokers' recollections of their first cigarette, in which current smokers report more positive recollections of this experience than current non-smokers [16-19].

While the studies briefly reviewed attempt to delineate factors that can mediate progression from initiation to habituation of smoking, a recent line of research postulates that this progression is essentially universal and is propelled by nicotine addiction that develops very rapidly [20-31]. In contrast to the studies reviewed above and to previous models of smoking progression [32,33], this line of research holds that one puff from a cigarette may be enough to get a teenager "hooked" on cigarettes. Researchers associated with the "hooked on nicotine" thesis have asserted that their findings carry imperative implications for smoking prevention policies. Scragg et al. [34], for example, concluded their study with the warning that "in light of the strength of the accumulated evidence, it would be irresponsible to withhold from youth a clear warning that experimentation with even one cigarette may initiate addiction. Legislation world-wide should aim to end the sale of single cigarettes and small packs, and ban the distribution of free samples of tobacco products" (p. 697). The purpose of the present article is to examine the validity of these far-reaching conclusions. We begin by summarizing some of the central studies in this research program, noting their major findings as well as major questions that these findings raise. We follow by critically examining the conceptual and empirical basis for the claims made by the "hooked on nicotine" program.

### Major findings and associated questions

A highly cited study by O'Loughlin et al. [28] reported the survey responses of 241 grade seven students who smoked "a puff or more" in the 3 months preceding the survey. Its findings were disturbing: Over half of the students who smoked only 1-2 cigarettes in their lifetime ("triers"), according to the study, have "lost autonomy" over their smoking. The findings reported in this study, however, invoke some puzzling questions. How can adolescents who smoked only one cigarette in their lifetime be claimed to have lost autonomy over their smoking? Presumably, the fact that they never smoked again testifies against such loss of autonomy. Just as inexplicable is the finding that over one third of the "sporadic smokers" in this sample reported "feeling nervous, anxious, tense on stopping." "Sporadic smokers" were defined as those who smoked at least one cigarette per year but less than one cigarette per month. How could such smokers have withdrawal symptoms "on stopping?" It would appear that, by definition, these responders were in a virtually permanent state of stopping.

According to O'Loughlin et al. [28], 13% of the "triers" believed they were mentally addicted and 11% that they were physically addicted to smoking. Assuming for a minute that these children could make valid judgments about the causes of their "symptoms" (as we shall discuss later, there is no reason to assume that), what can it mean when a teenager who smoked at most a couple of cigarettes in her lifetime perceives herself as mentally or physically addicted?

Similarly puzzling are the findings of a prospective study of 217 six-grade students from Massachusetts who have ever inhaled on a cigarette [24]. The study reported that 127 of these "inhalers" lost autonomy over their tobacco use, 10% having done so within 2 days and 25% having done so within 30 days of first inhaling on a cigarette; half had lost autonomy by the time they were smoking 7 cigarettes per month. These findings contradict those of a large body of studies of adult "chippers" [35], who "despite having smoked tens of thousands of cigarettes, show few signs of nicotine dependence" [36], p. 509). Moreover, DiFranza et al. [24] reported that tobacco dependence as defined by the ICD-10 was diagnosed as early as 13 days after the first inhalation. Eighty three of the inhalers (38.2%) developed ICD-10-defined tobacco dependence, half of whom by the time they were smoking only 46 cigarettes per month. According to the study, 25% of the participants were ICD-10 nicotine dependent when they were smoking only 8 or fewer cigarettes per month. These latter findings are perplexing. First, ICD criteria require that the symptoms "should have occurred together for at least 1 month or, if persisting for periods of less than 1 month, should have occurred together repeatedly within a 12-month period" [37], so how could the diagnosis be made 13 days following the first inhalation? And how could other symptoms required to make the diagnosis (e.g., withdrawal, tolerance, preoccupation with the substance, continued use despite harmful effects) develop in such a brief period?.

Gervais et al. [38] reported that "Mental addiction was concomitant with smoking a whole cigarette and sometimes occurred even before initiation, possibly reflecting high susceptibility to initiating tobacco use" (p. 260). Similar findings were reported in a more recent study [39], which assessed nicotine dependence symptoms among 10-12 year old children whether or not they smoked. Of 1488 never-smokers, sixty-nine (4.6%) reported at least one nicotine dependence symptom. According to these studies, then, adolescents can develop symptoms of nicotine addiction even when they have never smoked a puff, a proposition that seems counterintuitive.

Scragg et al. [34] reported the results of a very large survey (n = 96,156) of 14-15 year old students in New Zealand. The report concluded that "diminished autonomy" over smoking could be prompted by smoking a single cigarette: 46% of subjects who smoked less often than monthly reported one or more nicotine dependence symptoms. More than 25% of those who have smoked only one cigarette *in their lifetime *reported one or more symptoms. Nine percent of them reported that it was hard for them to keep from smoking in places where you are not supposed to. Most remarkably, 14% of those who smoked only one cigarette in their lifetime, and the same proportion of those who only smoked 1-2 puffs (in the 2004 survey), reported that they "have tried to quit but couldn't." It is unclear how one can to fail to quit if one has smoked at most a single cigarette in his or her lifetime. In discussing this finding, the authors acknowledge that this "may seem logically impossible." In an apparent attempt to resolve this logical difficulty, they report that "One author (JRD) has spoken with adolescents who claimed 'love at first puff', knowing from their reaction to their first cigarette that they would be smokers for life" (p. 696).

In sum, according to the "hooked on nicotine" line of research, adolescents can lose autonomy over their smoking after having smoked one puff in their lifetime and never having smoked again and can become mentally and physically addicted to nicotine even if they have never smoked a puff. Below, we examine the theoretical and empirical basis of these assertions. This examination shows that the conclusions of the "hooked on nicotine" research program are undermined by numerous conceptual and methodological shortcomings.

### Defining nicotine addiction as loss of autonomy

As the brief review above shows, many of the studies in the "hooked on nicotine" research program claim that adolescents can lose autonomy over their smoking behavior following a single puff from a cigarette and even following only second-hand exposure to cigarettes [39]. As this is the principal claim of this research program, we begin by describing the development of its conceptual and methodological tradition.

The first in the "hooked on nicotine" series of studies [20] concluded that "The first symptoms of nicotine dependence can appear within days to weeks of the onset of occasional use, often before the onset of daily smoking" (Abstract, p. 313). This was the first publication from the Development and Assessment of Nicotine Dependence in Youth (DANDY) study. The theoretical and methodological approach was the same one that would be used in later studies by this group: "Since the DSM-IV definition of nicotine dependence does not allow for the possibility that dependence might start before "prolonged heavy use", the DSM-IV criteria were not used in this study. Accordingly, subjects were not diagnosed as being nicotine dependent, or experiencing a "withdrawal syndrome" according to DSM-IV criteria. Rather, we report only on whether subjects report any individual symptoms that are associated with dependence" (p. 314).

In the next study with the same participants [22] 10 of the items used in the first study were modified to create the "Hooked on Nicotine Checklist" (HONC; see Table 1). The authors' definition of nicotine dependence was formalized in the framework of a novel "autonomy theory." According to autonomy theory, "the onset of dependence can be defined as the moment when an individual loses full autonomy over the use of tobacco. In philosophical terms, the loss of autonomy begins when discontinuing the use of tobacco is no longer an effortless exercise of free will." Moreover, "Based on the philosophical concept that an individual either has autonomy or does not" loss of autonomy was registered "if any of the 10 HONC items was endorsed at any time" (p. 399).

**Table 1:** The Hooked on Nicotine Checklist (adapted from Difranza et al. 2002b [22]).

1. Have you ever tried to quit but couldn't?
2. Do you smoke now because it is really hard to quit?
3. Have you ever felt like you were addicted to tobacco?
4. Do you ever have strong cravings to smoke?
5. Have you ever felt like you really needed a cigarette?
When you tried to stop smoking (or when you haven't used tobacco for a while)
6. Is it hard to keep from smoking in places where you are not supposed to, like school?
7. Did you find it hard to concentrate because you couldn't smoke?
8. Did you feel more irritable because you couldn't smoke?
9. Did you feel a strong need or urge to smoke?
10. Did you feel nervous, restless, or anxious because you couldn't smoke?

There are inherent problems with the conceptualization nicotine addiction as formulated by DiFranza et al. [22]. To begin with, the notion that loss of autonomy begins "when discontinuing the use of tobacco is no longer an effortless exercise of free will" is untenable. Many human behaviors are habitual and automatic rather than intentional and willful, so that both performing and discontinuing them is rarely "an effortless exercise of free will" [40,41]. One could replace "the use of tobacco" in the above definition with a range of behaviors from "brushing teeth in the morning" through "looking right and then left when crossing the street" to "saying 'bless you' when someone sneezes." By this criterion, then, humans have lost autonomy over most of their routine behaviors, which renders the criterion so non-specific that it loses any utility as a marker of addiction.

As shown above, the "hooked on nicotine" program holds that adolescents can lose autonomy over smoking after smoking a single puff in their lifetime and even when they have only been exposed to secondhand smoke. This leads to the paradoxical conclusion that one can lose autonomy over a behavior (in this case, smoking) that has never been performed. For the "hooked on nicotine" proponents this proposition may not be paradoxical, as they has consistently argued that all the criteria for nicotine addiction can be reduced to craving, which does not depend on consumption or any other behavior. For example, DiFranza et al. [26] have recently suggested that "Nicotine dependence measures such as days smoked per month, cigarettes smoked per day, time to first morning cigarette, a lot of time spent smoking, difficulty refraining, using more than intended, tolerance, use despite harm, prioritization and stereotypy of use [42-44], indirectly reflect the compulsion to smoke and/or the latency. A direct approach to determine/assess nicotine dependence would be to inquire about wanting, craving and needing and their respective latencies."

The suggestion that nicotine dependence can be reduced to craving is contradicted by converging lines of empirical evidence. First, craving is not specific to drugs. Many appetitive habits that do not involve drugs, such as eating [45,46], gambling [4,47] or the internet [48], are associated with craving levels that are just as powerful as those reported for the most addictive drugs [47]. As smoking combines (and therefore confounds) an appetitive behavioral habit and a drug, craving for smoking cannot be equated with craving for nicotine.

Second, in the case of smoking, craving is often dissociated from actual nicotine consumption or withdrawal. For example, religious Jews who do not smoke during the Sabbath [49] reported no craving on Saturday morning, following an overnight abstinence, but high levels of craving during a workday when they smoked ad lib. Similarly, non-daily smokers reported much higher craving levels on days that they smoked as compared to days that they did not smoke [50]. A study of flight attendants [51] who cannot smoke during the flight showed that craving was related to the time remaining to the end of the flight more than to the length of abstinence (and presumably, of nicotine withdrawal). These findings are inconsistent with the suggestion that nicotine addiction could be reduced to craving to smoke.

Moreover, a consequence of reducing nicotine dependence to subjective craving to smoke is that the results of the "hooked on nicotine" research program cannot be compared to results of studies that use the conventional, DSM or ICD conceptualization of nicotine dependence. In other words, this conception of addiction is so removed from the rest of the field's as to render the "hooked on nicotine" research program practically incommensurable with other relevant research. This problem is exacerbated by the methodology used to assess smoking dependence and related variables in this research program, to which we now turn.

### Employing lenient criteria for dependence

As noted earlier, most studies in the "hooked on nicotine" research program (e.g., [30,52] register "loss of autonomy," which signals the beginning of nicotine dependence, based on endorsement of a single HONC item. This approach has been recently criticized by Hughes and Shiffman [53], who noted three problems: "First, almost all disorders are syndromes that, by definition, are composed of multiple signs and symptoms. Second, requiring several signs and symptoms helps distinguish a clinically significant disorder. Setting a threshold so low as to classify almost all users as ''dependent'' risks blurring important distinctions among gradations of dependence (---). Third, endorsement of a single symptom can be unreliable and may reflect measurement error or other extraneous influences" (p. 1812). According to Hughes and Shiffman [53], "classifying smokers as ''hooked'' (i.e., fully dependent on nicotine) on the basis of endorsing a single marker of dependence is misleading in that it overdiagnoses and ignores further development of the severity of dependence" (p. 1812).

One exception to the problematic approach of registering "loss of autonomy" based on endorsement of any one of the HONC items was a study by DiFranza et al. [23]. As mentioned above, this prospective study of 217 six-grade students from Massachusetts who have ever inhaled on a cigarette reported that 83 of these "inhalers" (38.2%) developed ICD-10-defined tobacco dependence. Half of the participants met ICD-10 criteria by the time they were smoking only 46 cigarettes per month and 25% were nicotine dependent when they were smoking only 8 or fewer cigarettes per month and tobacco dependence as defined by the ICD-10 was diagnosed as early as 13 days after the first inhalation.

The import of these findings is undermined by the method used to establish ICD diagnoses in this study. The study used a 22-item interview to assess tobacco dependence symptoms. Three or more symptoms were required for a diagnosis, as required by the ICD criteria. The interview items used to assess these symptoms, however, were very lenient. For example, "Are you smoking more now than you planned to when you started?" was used to represent the criterion of difficulties in controlling tobacco-taking behavior in terms of its onset, termination, or levels of use. Neglect of alternative pleasures could be fulfilled by endorsing the item, "Do you find that you are spending more of your free time trying to get cigarettes?" The criterion of use despite harm could be fulfilled by answering affirmatively the question "Has a doctor or nurse told you that you should quit smoking because it was damaging your health?" Consequently, a participant who smoked two cigarette per week (but had planned on smoking only one), spent more time trying to get these two cigarettes than when he used to smoke only one per week and was told by the school nurse that smoking was bad for his health would earn in this study an ICD diagnosis of tobacco dependence. Findings based on such lenient criteria for tobacco dependence are of questionable significance, and again, cannot be compared to findings based on more conservative criteria.

### Relying on responders' causal attributions for physical and mental "symptoms"

In addition to the problems inherited in using a single item to assess "loss of autonomy," there are problems with the validity of the HONC items themselves. The principal problem is that most the items require participants to judge the causes of their own behaviors and feelings. This is particularly evident in regard to the items designed to assess nicotine withdrawal (the last 5 items in the HONC). The authors of the HONC were aware that many of the "symptoms" in this list may be unrelated to smoking, and attempted to resolve this potential confound. "Since symptoms associated with nicotine withdrawal, such as irritability, can have other causes these symptoms were counted only if subjects attributed them to nicotine withdrawal" [20], p. 317). This does not resolve the problem, however, it has been established for more than three decades now that such causal attributions have very limited validity. In their classic paper, "Telling More Than We Can Know," Nisbett and Wilson [54] demonstrated that when people provide introspective reports on the causes of their behavior, what they really are doing is making reasonable inferences about what the causes must have been. Moreover, Nisbett and Wilson [54] and others [55] showed that a major source of such *post hoc *causal inferences is culturally-provided theories. As nicotine addiction is a widely accepted theory for why people smoke, responders would be likely to perceive themselves as addicted to nicotine and to attribute "symptoms" such as lack of concentration and irritability to nicotine withdrawal, especially if this particular attribution is suggested by the survey items.

None of the articles we reviewed acknowledged the difficulty inherent in taking participants' causal attributions at face value. Some even assume that participants can accurately attribute their enjoyment of cigarettes to nicotine: "Several of our subjects seemed to describe a phenomenon akin to "love at first sight", sensing immediately that nicotine had a powerful influence on them" [[20], p. 317]. The practice of taking such causal attributions at face value is endemic to the "hooked on nicotine" research program and compromises the validity of the majority of its studies. Most significantly, it undermines the validity of the findings concerning self-reported physical and mental addiction to smoking.

Gervais et al. [38] examined "milestones related to symptoms of nicotine dependence." Among these milestones were "Time of first self-report of physical addiction: survey date on which the participant first responded "a little," "quite" or "very" to the question "How physically addicted to smoking cigarettes are you?"" An identical item was used to record the milestone of first self-report of mental addiction. Similar questions were employed in other studies by this group [28,38,39,56,57]. According to Gervais et al. [38], "These items were developed based on earlier qualitative work in which adolescents were asked to describe their experiences of nicotine dependence and were able to distinguish between what they perceived to be mental and physical addiction."

The cited study [27] was conducted to "explore adolescent smokers' understanding and their physiological and psychological experience of addiction to nicotine." The researchers used focus groups of teenagers who smoked in which they asked them, among other aspects of their experience, about addiction and loss of autonomy. The claim that participants could validly report physical addiction to nicotine was based on the observation that "When asked what exactly it was they were addicted to, participants readily answered that it is the nicotine in cigarettes." Clearly, the responders had no way of knowing this for a fact and their ready answer only proves that they believed that smoking was driven by nicotine. Additionally, some participants "described fairly specific physical symptoms." These "fairly specific" symptoms included "feeling of lack, or emptiness: 'feelings of emptiness, like an empty spot in here' (points to chest)---sensations in other parts of their bodies such as 'in your blood; in your head' (i.e., a physical sensation in the head); 'like being hungry'. Others had trouble detecting whether the feeling was more in their bodies or their minds: 'All of it, it's everything'; 'I don't know what the physical feeling of being addicted is'; 'I don't know how it feels in my body. I think I can only feel it in my head'; 'you feel it in both your head and your body'" (p. 205). So in fact, the study by O'Loughlin et al. [27] demonstrates only that the participants shared the belief that nicotine is the source of their addiction. The study does not provide any basis for the statement that young smokers can validly recognize physical addiction and distinguish it from mental addiction. Moreover, the claim that self-perceived mental and physical addiction can be validly distinguished by participants is inconsistent with the fact that the two measures are highly correlated.

**Figure 1:**
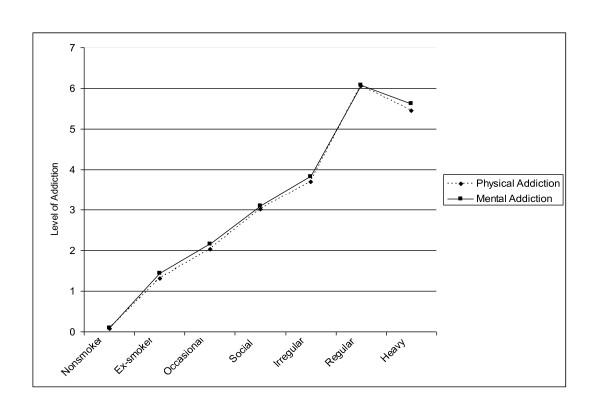
**The relationships of smoking status to self-reported physical addiction and mental addiction (Adapted from Figure 2 in Okoli et al. **[56]).

According to Okoli et al. [57], for example, "perceived mental and physical addiction were modestly correlated (Spearman's rho = .64, p < .001)." A correlation of .64 is hardly "modest" - in fact, considering that single items are not very reliable, it suggests a very considerable overlap between the two items. The overlap between the measures is also evident in studies which report their correlations with other measures. In Richardson et al. [58], for example, the patterns of correlations between mental and physical addiction and other measures of dependence are essentially identical (see [58] - Table four). An especially vivid illustration of the overlap between the two measures is provided in a study by Okoli et al. [56], in which the relationships of smoking status to physical addiction and to mental addiction are presented graphically side by side (Figure two in [56] - see Figure 1). The two curves are identical, as can clearly be seen from Figure two, in which we superimposed the values for the two curves. The identity of the two curves strongly suggests that self-reported physical addiction cannot be distinguished from self-reported mental addiction.

### Biased coding and interpretation of the data

In addition to the methodological flaws noted above, several of the studies we reviewed were marred by biased coding and interpretation of the data. For example, Okoli et al. [57] examined the relationship between self-reported physical and mental addiction to tobacco and perceived susceptibility to smoke in the future. Participants were asked how physically addicted and how mentally addicted to tobacco they were right now. Responses were rated on 10-point scales with 0 = "not at all addicted" and 10 = "very addicted." However, because of the severely positively skewed distributions of these addiction measures, "and for ease of conceptual interpretation, individuals selecting "0" were coded as '0 = no' and individuals selecting greater than "0" were coded as '1 = yes'." Thus, a respondent who rated his level of dependence as "1" on a scale of 0-10 was categorized as perceiving himself as addicted to tobacco, which is a rather distorted interpretation of this response.

Similarly, susceptibility to smoking was assessed by asking participants how likely it is that they will ever smoke in the future, with response choices: 'very likely,' 'somewhat likely,' 'rather unlikely' and 'very unlikely.' In creating the categories of 'susceptible' and 'unsusceptible,' the authors "applied a strict criterion of limiting the 'nonsusceptible' category to only those who were 'very unlikely'." In other words, responders who said they were 'rather unlikely' to ever smoke in the future were treated as if they perceived themselves as susceptible to future smoking, which again distorts the meaning of this response. This is particularly puzzling considering that in their previous study using the same scale [56], the response 'rather unlikely' was coded as 'non-susceptible."

The findings of Okoli et al. [57] are presented in a way that further distorts the actual data. The study is titled "Non-smoking youths' "perceived" addiction to tobacco is associated with their susceptibility to future smoking." Susceptibility, however, is just as "perceived" as addiction in this study. The study does not show that perceived addiction predicts actual susceptibility (future use). It only shows a correlation between two subjective responses, one of which (perceived addiction) may be of doubtful validity, especially in naïve participants. Adding to that the fact that both responses were re-coded in a way that distorts their original meaning limits any conclusions that can be drawn from this study.

In the study of Gervais et al. [38] mentioned above, "Time of first withdrawal symptom" was defined as the survey date on which the participant first responded "rarely," "sometimes" or "often" to the question "Now think about the times when you have cut down or stopped using cigarettes or when you haven't been able to smoke for a long period (like most of the day). How often did you experience feeling a strong urge or need to smoke?" The response "rarely" to this question was coded as confirmation of withdrawal, which again distort the meanings of this response and undermines any conclusions that can be drawn from it.

Some of the studies suffer from an undetected statistical bias. For example, to support their theory that the number of cigarettes smoked has a critical impact on getting hooked, Scragg et al. [34] present a figure (Fig. one in [34]) that shows a negative linear relationship between the number of cigarettes ever smoked and the probability of being currently abstinent. According to the article, "Fig. one is rather ominous in its depiction of the relationship between early tobacco use, the loss of autonomy, and the dwindling prospects for early cessation. Beginning with the first, each cigarette appears to increase the likelihood that autonomy will be lost, and to decrease the likelihood of quitting" (p. 697). The inference that the number of cigarettes ever smoked is causally related to the ability to quit is false, however, as the two figures are not independent. The chance of being categorized as a current smoker in the survey was higher for those who smoked more in their lifetime simply as a statistical fact. If one participant has smoked 100 cigarettes over her lifetime and another only two, there was a much higher likelihood that the former participant would have smoked at least one of her cigarettes during the survey period, which would earn her the label of "current smoker."

Finally, some of studies contain biases that seem to stem from confusion in regard to the mechanisms that are believed to underlie smoking dependence. For example, in the study of Gervais et al. [38] discussed above, another "milestone" related to nicotine dependence was "Time of first symptom of tolerance: survey date on which the participant first responded "a bit true" or "very true" to the statement "Compared to when I first started smoking, I can smoke much more now before I start to feel nauseated or ill." This item misses the point, as addiction is supposed to be driven by tolerance to the *reinforcing *effects of drugs, not to their aversive or negative effects. The mechanism by which tolerance leads to increased drug use is that the user no longer receives these desired effects, as DiFranza and Wellman [59] specifically posit in their own theory of nicotine addiction. Tolerance to the negative effects of drugs enables using more of the drug, but does not motivate increased use [60].

## Conclusion

The "hooked on nicotine" research program addresses the very important and timely issue of adolescent smoking. This review of the "hooked on nicotine" research program suggests, however, that its findings concerning the speed and ease by which adolescents can become addicted to smoking are invalidated by major conceptual and methodological flaws. These flaws include an untenable and idiosyncratic conceptualization of addiction which is incommensurable with the rest of the field's, basing the assessment of dependence on a single item or on extremely lenient criteria and relying on participants' causal attributions in regard to their subjective states, including self-reported mental and physical addiction. While these methodological limitations are sometimes noted, they are generally downplayed and do not affect the decisiveness of the conclusions. Interpretation of the findings is often biased and obvious caveats and alternative explanations for the data are often ignored. These problems undermine the contribution of the "hooked on nicotine" research program to the understanding of the nature and development of tobacco smoking in adolescence. Further research in this important area should consider the conceptual and methodological problems noted in this review in order to produce more reliable evidence regarding the initiation and progression of smoking in adolescents.

## Competing interests

RD and HF have received fees for consulting to lawyers working with tobacco companies. However, all their research, including this review, is supported exclusively by academic funds.

## Authors' contributions

RD and HF reviewed the literature and wrote the paper together. Both authors contributed to and have approved the final manuscript.
